# Incidence and epidemiological features of dengue in Sabah, Malaysia

**DOI:** 10.1371/journal.pntd.0007504

**Published:** 2020-05-11

**Authors:** Amanda Murphy, Giri Shan Rajahram, Jenarun Jilip, Marilyn Maluda, Timothy William, Wenbiao Hu, Simon Reid, Gregor J. Devine, Francesca D. Frentiu

**Affiliations:** 1 Mosquito Control Laboratory, QIMR Berghofer Medical Research Institute, Brisbane, Australia; 2 School of Biomedical Sciences, and Institute for Health and Biomedical Innovation, Queensland University of Technology, Brisbane, Australia; 3 Queen Elizabeth Hospital, Ministry of Health Malaysia, Kota Kinabalu, Malaysia; 4 Infectious Disease Society of Kota Kinabalu-Menzies School of Health Research Clinical Research Unit, Kota Kinabalu, Malaysia; 5 Sabah Department of Health, Ministry of Health Malaysia, Kota Kinabalu, Malaysia; 6 Gleneagles Kota Kinabalu Hospital Sabah, Kota Kinabalu, Malaysia; 7 School of Public Health and Social Work, Queensland University of Technology, Brisbane, Australia; 8 School of Public Health, University of Queensland, Brisbane, Australia; Chinese Center for Disease Control and Prevention, CHINA

## Abstract

In South East Asia, dengue epidemics have increased in size and geographical distribution in recent years. We examined the spatiotemporal distribution and epidemiological characteristics of reported dengue cases in the predominantly rural state of Sabah, in Malaysian Borneo–an area where sylvatic and urban circulation of pathogens are known to intersect. Using a public health data set of routinely notified dengue cases in Sabah between 2010 and 2016, we described demographic and entomological risk factors, both before and after a 2014 change in the clinical case definition for the disease. Annual dengue incidence rates were spatially variable over the 7-year study period from 2010–2016 (state-wide mean annual incidence of 21 cases/100,000 people; range 5-42/100,000), but were highest in rural localities in the western districts of the state (Kuala Penyu, Nabawan, Tenom and Kota Marudu). Eastern districts exhibited lower overall dengue rates, although a high proportion of severe (haemorrhagic) dengue cases (44%) were focused in Sandakan and Tawau. Dengue incidence was highest for those aged between 10 and 29 years (24/100,000), and was slightly higher for males compared to females. Available vector surveillance data indicated that during large outbreaks in 2015 and 2016 the mosquito *Aedes albopictus* was more prevalent in both urban and rural households (House Index of 64%) than *Ae*. *aegypti* (15%). Demographic patterns remained unchanged both before and after the dengue case definition was changed; however, in the years following the change, reported case numbers increased substantially. Overall, these findings suggest that dengue outbreaks in Sabah are increasing in both urban and rural settings. Future studies to better understand the drivers of risk in specific age groups, genders and geographic locations, and to test the potential role of *Ae*. *albopictus* in transmission, may help target dengue prevention and control efforts.

## Author summary

In order to combat the rising regional incidence of dengue in South East Asia, incidence patterns must be better characterised within different ecological settings. We conducted the first retrospective analysis of dengue epidemiology in the predominantly rural state of Sabah, Malaysia, where both urban and sylvatic transmission cycles exist. Human notification data over a 7-year period were reviewed and spatiotemporal and demographic risk factors identified. We found that: 1. While urban habitats do play a role in mediating the spread of dengue in Sabah, cases from both urban and rural localities contributed equally to dengue outbreaks; 2. Human demographic risk factors included being aged between 10 and 29 years, and being male; 3. Cases of severe dengue were more common in the eastern districts of the state–almost half of severe dengue cases were reported from Sandakan and Tawau; and 4. The presence of *Aedes albopictus* in and around the majority of urban and rural case households, often in the apparent absence of *Ae*. *aegypti*, suggests that its role in transmission requires further investigation. This study emphasises that increasing incidence of dengue in urban South East Asia is also mirrored in more rural areas, and suggests a need for control strategies that address both urban and rural dengue risk.

## Introduction

Dengue is the most rapidly spreading vector-borne disease in the world, and the most prevalent arboviral disease of humans [1]. Now endemic in more than 100 countries, the disease causes an enormous burden on communities and health care systems in tropical and sub-tropical regions [2]. The causative agent of dengue is dengue virus (DENV), transmitted between humans by *Aedes* mosquitoes across a range of domestic and sylvatic environments. Urban expansion, human migration, travel and trade have facilitated an increasing number of infections, primarily in Asia, Africa and North and South America [3, 4]. The Americas and Asia have been identified as high risk zones due to the presence of dense populations of humans and vectors and climate suitability, with Asia in particular having a disproportionate burden (up to 70%) of global infections [1]. The number, severity and geographic distribution of dengue epidemics has increased in South East Asia since the 1950s, when the first cases of the severe (haemorrhagic) form of dengue were identified during epidemics in Thailand and the Philippines [3, 5]. Outbreaks continued throughout South East Asia during the 1960s, including Vietnam, the Philippines, Singapore, Malaysia and Thailand, with Malaysia’s first severe dengue outbreak recorded in Penang in 1962 (61 cases and 5 deaths reported) [6].

In Malaysia, regular outbreaks have occurred since the 1960s with the first major, nation-wide outbreak of 1,487 cases and 54 deaths recorded in 1973 [7]. Steep increases in case numbers began to occur from the late 1980s when incidence rates rose from 9 cases/100,000 in 1988 to 123 cases/100,000 in 1998, reaching a total of 27,381 reported cases in 1998 [8]. These increases occurred alongside Malaysia’s rapid population and infrastructure growth (rising from 13.7–23.3 million people between 1980 and 2000), which facilitated the spread of dengue through the unintended creation of new vector breeding sites [9, 10]. This trend continued into the 21^st^ century, with a 7-fold increase in case numbers between 2000 and 2010, when case numbers reached 46,171 (incidence rates of 30 and 162/100,000, respectively) [11]. In 2014, the largest ever outbreak was recorded, with 108,698 cases (incidence rate of 361/100,000, with 215 deaths) [12, 13]. This coincided with the introduction of a new national dengue case definition in that same year, requiring all notifications to be laboratory-confirmed by a diagnostic test (either NS1 and/or IgM/IgG serology) [14]. While the majority of reported cases have been concentrated in the highly urbanized states of Selangor, Kuala Lumpur and Johor, located on the Malaysian peninsula (together comprising 68% of cases between 2012 and 2016), increases have also been recorded in more rural states [15, 16]. Few eco-epidemiological studies have explored the factors driving incidence rates in rural parts of the country; however, seroprevalence and vector surveillance studies suggest that infection risk has become comparable in rural and urban areas alike [16–19].

As with many South East Asian countries, the drivers of dengue disease in Malaysia are multi-faceted, and encompass characteristics of, and interactions between, virus, vectors, hosts and their environments. These include viral virulence and human biological factors; climate factors, including high temperature, relative humidity and increased rainfall; human movement and behaviour; and economic and infrastructure development [9, 20]. These may alter human susceptibility to infection, promote mosquito breeding and/or increase interactions of viruses, vectors and hosts. The Malaysian Ministry of Health has reported inappropriate waste disposal to be a major dengue prevention challenge, with polystyrene food containers, plastic bottles and tyres contributing the highest percentage of artificial mosquito breeding sites [21]. Mosquito breeding and human movement facilitate the ongoing circulation of all four human DENV serotypes across the country, although circulation patterns are distinct between states [22, 23]. In addition, sylvatic DENV serotypes detected from human cases on the island of Borneo suggest a greater diversity of viruses in some habitats [24–26] and the potential for sylvatic viruses to enter human transmission cycles [27].

The Malaysian states of Sabah and Sarawak, in Malaysian Borneo, report lower incidence rates than mainland Malaysia (together, 5% of all cases between 2012–2016) and patterns of transmission in these states are not well characterised [15]. Rapidly developing urban areas are located in close proximity to disturbed forest environments, facilitating interactions among DENV vectors and hosts, and increasing potential risk of spill-over of sylvatic pathogens to human populations [24, 28]. Sabah has high rates of forest loss, with monocultures of rubber and palm plantations estimated to cover 36–56% of the land area [28, 29], and reports the highest incidence of the sylvatic malaria parasite *Plasmodium knowlesi* [30]. Increased incidence rates of *P*. *knowlesi* malaria in Sabah have been linked to deforestation and land use changes, which lead to increased contact between its *Anopheline* vectors, primate hosts and humans [31, 32]. Given the marked environmental change occurring in Sabah, and the increase in dengue cases noted in recent years [15, 26], it is essential from a public health perspective to understand current disease patterns and their associated risk factors. Our study describes recent spatial and temporal trends in dengue incidence, and some potential demographic and entomological risk factors from this understudied region of South East Asia.

## Methods

### Ethics statement

This study was approved by the Medical Research and Ethics Committee (MREC), Ministry of Health Malaysia; and the Human Research Ethics Committee (HREC) of the QIMR Berghofer Medical Research Institute, Brisbane, Australia. All human case data analysed were anonymized.

### Study site

**Fig 1:**
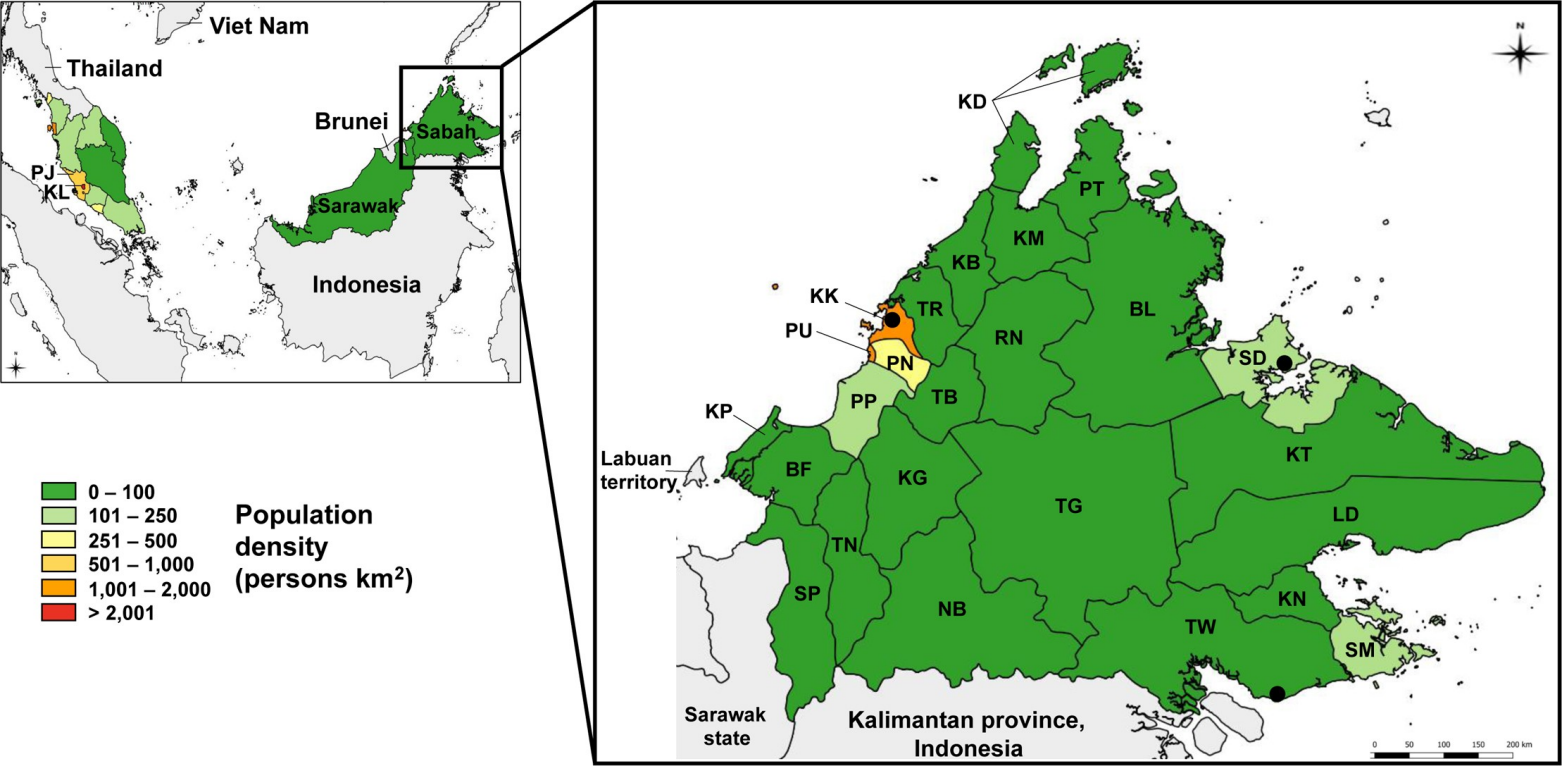
Map of Malaysia and Sabah state. Peninsula Malaysia and Malaysian Borneo are shown, along with the 13 Malaysian states and 3 territories: Kuala Lumpur (KL), Putrajaya (PJ) and Labuan. States are coloured according to their population density, expressed as number of people per square km. The island of Borneo includes the Malaysian states Sabah and Sarawak, and is also shared by the country of Brunei and the Indonesian province of Kalimantan. *Inset*: Sabah state, showing its 25 districts: Beaufort (BF), Beluran (BL), Keningau (KG), Kinabatangan (KT), Kota Belud (KB), Kota Kinabalu (KK), Kota Marudu (KM), Kuala Penyu (KP), Kudat (KD), Kunak (KN), Lahad Datu (LD), Nabawan (NB), Papar (PP), Penampang (PN), Pitas (PT), Putatan (PU), Ranau (RN), Sandakan (SD), Semporna (SM), Sipitang (SP), Tambunan (TB), Tawau (TW), Tenom (TN), Tongod (TG), Tuaran (TR). The three largest cities in the state are indicated by black points: the capital city Kota Kinabalu, Sandakan and Tawau.

The Malaysian state of Sabah lies at the most north-eastern tip of the island of Borneo. It borders the Malaysian state of Sarawak and the Indonesian province of Kalimantan. The climate is tropical; there is high humidity and year-round rainfall, which increases between November and March. Sabah has a geographical area of 73,904 km^2^ and is divided into 25 districts [29]. Of Malaysia’s 13 states and 3 territories, Sabah’s population density is second lowest in the country (44 people/km^2^), after Sarawak (20 people/km^2^) (Fig 1). Sabah also has a relatively low urban population proportion compared with other Malaysian states (54% of Sabah’s population live in urban areas, compared with 100% in Kuala Lumpur and Putrajaya) [15]. Within Sabah, Kota Kinabalu district has the highest population density (1,397 people/km^2^, with almost 500,000 people), where the capital city of the same name is located.

### Epidemiological data

State-wide data from monthly notified cases of dengue between the years 2010 and 2016 were obtained from the Sabah State Department of Health (Jabatan Kesihatan Negeri, Sabah), Malaysian Ministry of Health. The data were collected as part of routine public health monitoring of dengue case reports from health facilities in Sabah. Prior to 2010, these data were not available in disaggregated and electronic format. Within the data set made available for this study, epidemiological variables included the age and gender of each case, the district and locality (urban or rural) of each case residence, disease severity and outcome (survival or death), and diagnostic tests performed (IgG, IgM and/or NS1). In our dataset, locality status was available for the 6-year period from 2011–2016. Locality of case residence represented the smallest residential geographical unit used by the Malaysian Department of Statistics, and was allocated based on home address. Designation of locality status (urban or rural) was according to the Malaysian Department of Statistics definitions of urban and rural, where urban localities are gazetted census areas with 10,000 people or more, and ≥60% of the working population (≥15 years) engaged in non-agricultural activities [15]. In our dataset, locality status was recorded for case residences from 2011 onwards. Population and demographic data for Sabah districts were obtained from the Malaysian Department of Statistics for the year 2010. Estimated population numbers for each age, gender and district were then calculated for each subsequent year using published annual population growth rates [10]. These estimated resident population numbers were used to calculate mean annual incidence rates.

During the study period, clinical cases were identified using World Health Organization (WHO) guidelines using clinical symptoms and/or positive NS1 or serology (presence of IgM or IgG) [33]. Diagnosis of severe dengue was based on identifying more serious symptoms including severe plasma leakage, severe haemorrhage and severe organ dysfunction [34]. From 2014 onwards, Malaysian national notification guidelines were modified, in line with WHO advice, to require a positive laboratory diagnostic test (either NS1 and/or IgM/IgG serology) in addition to the presence of clinical symptoms, and case notification within 24 hours of diagnosis [14, 35]. Therefore, the majority of cases prior to 2014 were clinically diagnosed (with 30–50% per year confirmed by laboratory tests in our dataset), while cases from 2014–2016 were 100% laboratory confirmed.

### Entomological data

Entomological surveillance data (number of larvae, mosquito species identified) were generated from active surveillance of potential aquatic habitats, primarily water-holding containers, indoors and outdoors around 719 case residences inspected during the 2015–16 outbreaks. Surveys were carried out by the local public health authority who designated 255 (36%) of inspected residences as urban, and 437 (61%) as rural. No rural or urban designation recorded for a further 27 (4%) of houses. Where mosquito larvae were found in or around a case household, samples were taken to local public health laboratories for species identification. The presence or absence of one or more species per household was recorded, and the House Index (HI) was calculated as the proportion of houses infested with larvae and/or pupae [36].

### Data analysis

We assessed seasonal characteristics of the temporal distribution of cases using a seasonal trend decomposition procedure in SPSS software. The procedure is based on the Census Method I, otherwise known as the ratio-to-moving-average method where time series data are separated into a seasonal component, a combined trend and cycle component, and an "error" or irregular component [37]. The seasonal component is then isolated from the overall and irregular trends through a multiplicative model. Seasonal decomposition analysis was applied to monthly dengue case numbers across the 7-year period to examine the seasonal trends of case notifications across Sabah.

Annual, monthly and mean incidence of dengue were calculated using the number of notifications per month and Sabah population estimates based on the 2010 Malaysian census. Incidence rates were standardized for age and gender by adjusting population numbers for these variables by their relative district-level population proportions. Ages of cases were grouped into four categories to broadly separate young children from older children and adults (0–9, 10–29, 30–49 and ≥50). Differences between means were determined by Kruskal-Wallis or Mann-Whitney statistical tests using SPSS Statistics software (SPSS, IBM New York USA; version 23). Statistical significance was set at p<0.05. Maps of Malaysia and Sabah dengue cases and incidence were created using ArcGIS (Esri Redlands USA; version 10.5.1).

We assessed overall and annual trends of rural versus urban cases (locality status) at the state-wide level for a 6-year period where this variable was available (2011–2016). This included a total of 9,791 cases. Of these, 756 (7.7%) cases were missing a designated locality status (rural or urban), due to incomplete data entry. We excluded these from rural and urban incidence calculations. For the remaining 9,035 cases, we calculated the total proportions and incidence rates for urban and rural cases, using population projections calculated from state-wide rural-urban population data published in 2010 [10]. At district level, we calculated annual and overall proportions of rural and urban cases per district. Where cases with unspecified localities were included in analyses (Tables 1 and 2 and S1 Table), the proportion of unspecified localities were indicated. Annual and overall relative risks (RR) of dengue for each individual district were calculated using:

$$RR=\frac{Observed\;incidence\;rate}{Expected\;incidence\;rate}$$

where the expected incidence rate for each district is based on the mean rate for the state multiplied by the population of each district. A RR value >1 indicates increased incidence of dengue in that location compared to the expected (mean) incidence, and a value <1 indicates lower than expected dengue incidence.

**Table 1:** Summary of population and dengue burden across Sabah, 2010–2016.

District	Human population (2010)	Population density (people/km^2^)	Proportion of cases from urban localities*	Mean annual incidence (per 100,000)	Severe dengue mean annual incidence (per 100,000)	Overall relative risk	Number of dengue deaths
Beaufort	66,406	38	0.06	49	0.2	0.9	0
Beluran^#^	106,632	14	0.14	34	0.9	0.8	1
Keningau	177,735	50	0.11	57	0.2	0.9	0
Kinabatangan	150,327	23	0.04	19	0.0	0.3	0
Kota Belud	93,180	67	0.02	52	0.7	1.4	0
Kota Kinabalu	462,963	1,315	0.85	57	0.2	1	5
Kota Marudu	68,289	36	0.02	79	0.4	2.1	1
Kuala Penyu	19,426	43	0.06	161	0.0	3.5	0
Kudat	85,404	66	0.50	52	0.5	1.8	1
Kunak	62,851	55	0.62	33	1.3	1	0
Lahad Datu	206,861	28	0.42	45	0.8	1.3	2
Nabawan^^^	32,309	5	0.00	88	0.0	1.6	0
Papar	128,434	103	0.20	31	0.0	0.6	0
Penampang	125,913	270	0.51	74	0.6	1.3	1
Pitas	38,764	27	0.29	36	0.0	0.8	0
Putatan**	55,864	1,397	0.32	73	0.0	1	2
Ranau	95,800	26	0.09	37	0.1	0.7	1
Sandakan	409,056	180	0.80	57	1.0	1.1	4
Semporna	137,868	120	0.53	46	1.1	1	2
Sipitang	35,764	13	0.14	56	0.0	1.2	0
Tambunan	36,297	27	0.00	37	0.0	0.8	0
Tawau	412,375	67	0.73	33	0.8	0.8	5
Tenom	56,597	13	0.12	84	0.7	1.5	0
Tongod**	36,192	4	0.05	22	1.0	0.4	0
Tuaran	105,435	90	0.26	56	0.4	1.3	0
**Total**	**3,206,742**	**43**	**0.48**	**50**	**0.5**		**25**

* Rural urban locality data was included from 2011–2016; overall proportions were 0.48 urban, 0.44 rural and 0.08 unspecified.

^#^ Beluran was formerly known as Labuk Sugut.

^^^ Nabawan was formerly known as Pensiangan.

** Putatan and Tongod districts only commenced notifications in 2012.

**Table 2:** Mosquito larvae species collected from case residences in Sabah during 2015–2016.

Locality	Total number of dengue cases in 2015–2016	No. of case residences inspected	No. of larvae-positive case residences (all species)	House Index (HI)	Number of larvae-positive residences with specific species present (HI)				
					*Ae*. *aegypti*	*Ae*. *albopictus*	*Ae*. *aegypti* & *Ae*. albopictus^*^	*Culex* spp.	Undetermined
Urban residences	3,157	255	206	0.81	47 (0.23)	142 (0.69)	7 (0.03)	6 (0.03)	4 (0.02)
Rural residences	2,824	437	388	0.89	33 (0.09)	225 (0.58)	4 (0.01)	26 (0.07)	100 (0.26)
Locality unspecified	565	27	24	0.89	3 (0.13)	16 (0.67)	0 (0.0)	1 (0.04)	4 (0.17)
**Total**	**6,546**	**719**	**618**	**0.86**	**83 (0.13)**	**383 (0.62)**	**11 (0.02)**	**33 (0.05)**	**108 (0.17)**

HI = proportion of residences positive for mosquito larvae, calculated as number of residences with larvae/number of residences inspected.

* Both species found breeding together in one household.

Species were undetermined if the larvae failed to survive to adults to be identified, or if identification was pending/incomplete.

## Results

### Temporal trends across the state

**Fig 2:**
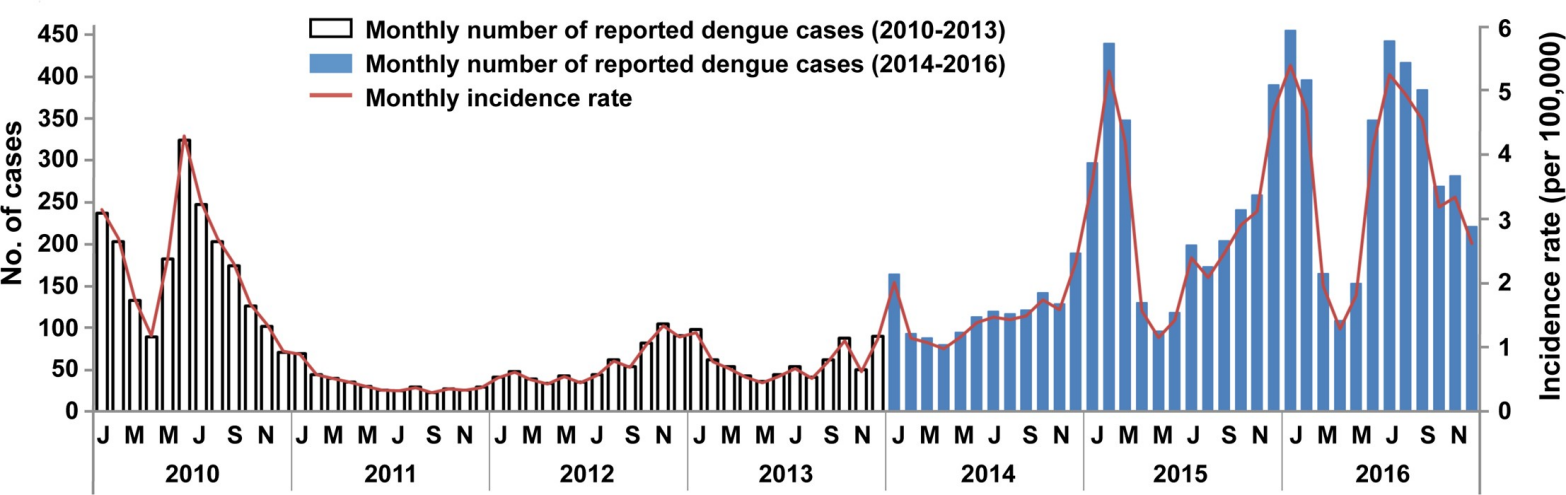
Temporal pattern of dengue in Sabah, 2010–2016. The monthly number of reported dengue cases per year are shown (primary vertical axis), and the corresponding monthly incidence rate (secondary vertical axis). Grey bars indicate the years 2010–2013 where case diagnoses were predominantly clinically-based (with or without laboratory confirmation), and blue bars indicate notifications following the 2014 change in case definition, where all cases were laboratory confirmed.

A total of 11,882 dengue cases were notified in Sabah during the 7-year study period, with 25 deaths. Cases were notified year-round, with outbreaks commonly occurring in the second half of the year between July and December, sometimes continuing into January and February (Fig 2). Seasonal decomposition analysis showed that, on average, notifications peaked each January, with the highest risk period being between November and March. The typical off-peak months were between April-June (S1 Fig).

**Fig 3:**
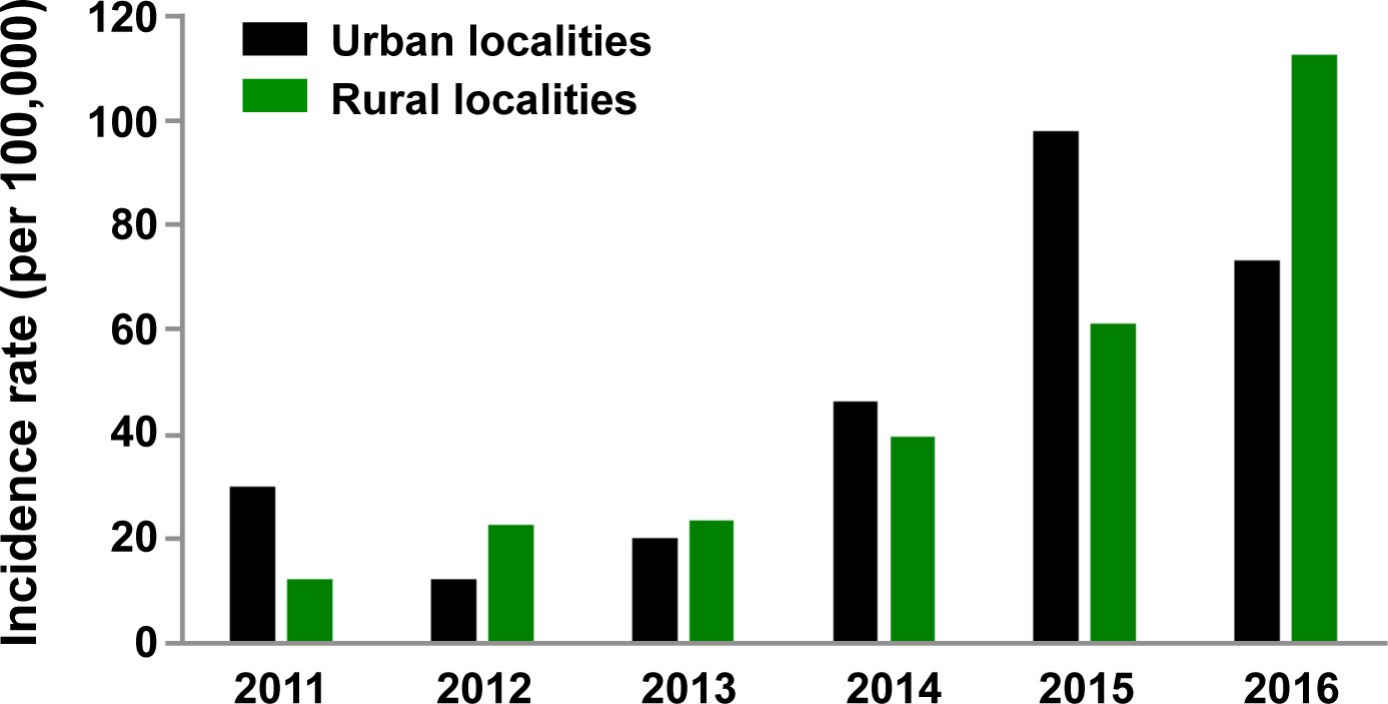
State-wide annual incidence of dengue in rural and urban localities, 2011–2016. Annual incidence rates (per 100,000 population) across the state are shown for cases residing in either urban or rural localities, over a 6-year period.

Outbreaks varied in magnitude between years, with the largest outbreaks in 2010 and from 2015–2016 (Fig 2). Between 2010–2013, the mean annual incidence was 13 cases/100,000 and this increased to 32 cases/100,000 between 2014–2016, coinciding with the change in national notification guidelines. The mean state-wide annual incidence rate across the 7 years was 21 cases per 100,000 people (median 18/100,000). For the 6-year period (2011–2016) where locality data were available, state-wide mean annual incidence of dengue in urban localities was 44/100,000 versus 47/100,000 for rural localities. Annual rates of dengue in urban and rural localities often contributed similarly to the overall burden, despite annual variations in incidence (Fig 3). Their relative contributions to dengue incidence were also roughly equivalent both before and after the 2014 revised case definition.

### Demographic trends

**Fig 4:**
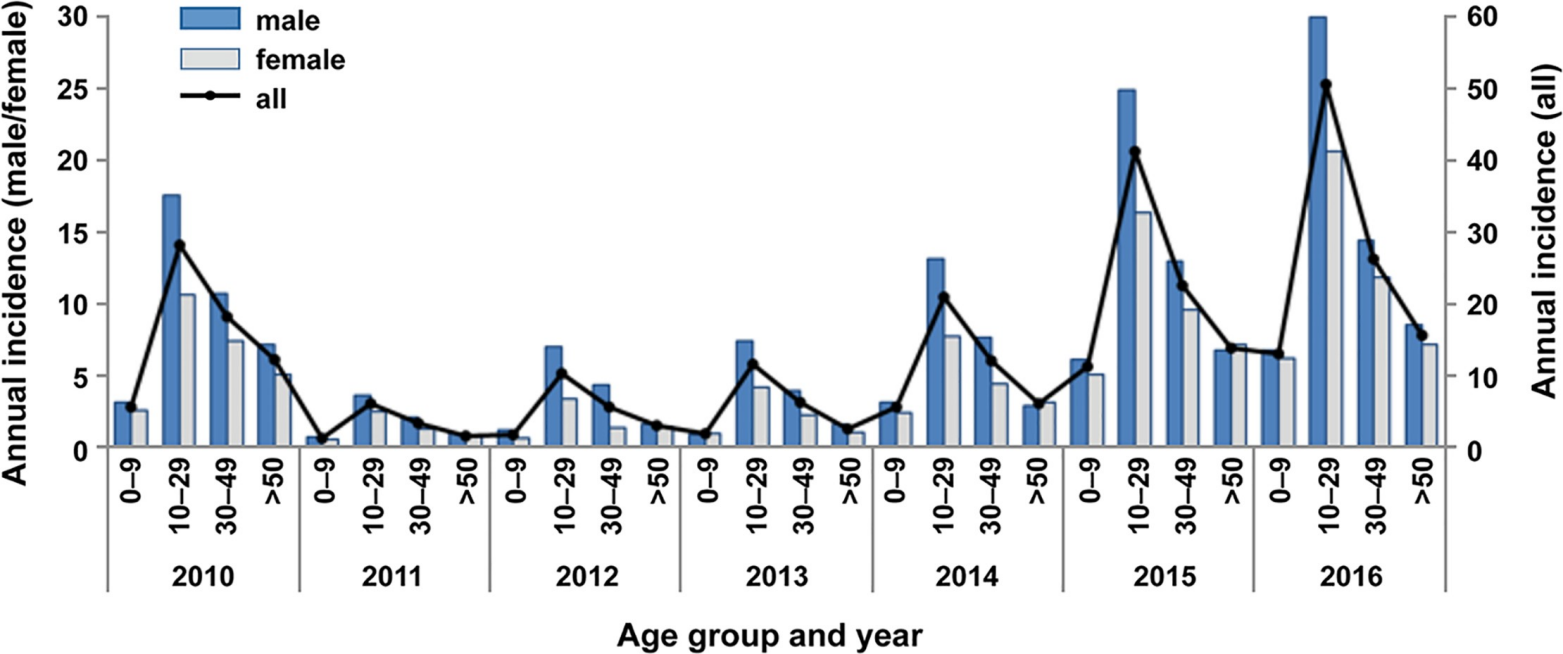
Incidence of dengue in Sabah by age group and gender, 2010–2016. Age- and gender-adjusted incidence rates (per 100,000 population) across Sabah during the 7-year period are shown for males, females and for both genders.

Analyses of demographic trends across Sabah indicated a slightly higher proportion of male dengue cases (60%) than females (40%); however, mean annual incidence rates were not significantly different between genders across the 7 years (29/100,000 for males and 20/100,000 for females, Mann-Whitney U = 19, p = 0.535), nor within different age groups (U = 321, p = 0.241) (Fig 4). This was relatively consistent across all Sabah districts; however, above-average proportions of male cases were observed in Tongod and Kinabatangan (75% and 65% male cases, respectively).

For both genders, nearly half of the case burden (47%) was borne by those aged between 10 and 29 years (mean annual incidence of 24 cases/100,000), followed by 30–49 years (26% of total cases, and 13 cases/100,000). The median age of all notifications was 25. After standardising for age and gender, incidence rates were found to be significantly different between age groups (Kruskal-Wallis H = 9.046, p = 0.029), with pairwise comparisons identifying a significant difference between the 0–9 and 10–29 groups only (Mann-Whitney U = 5.0, p = 0.011). The lowest proportion of notifications occurred below 10 years of age (mean annual rate of 6 cases/100,000), followed by those ≥50 years of age (8 cases/100,000). The incidence trends for gender and age groups remained consistent both before and after the 2014 case definition change.

### Spatial trends across districts

District-level incidence rates were highly variable each year, with a mean annual rate of 50 cases/100,000 (range 19-161/100,000) across the 7 years (Table 1, S2 Fig). High annual variability meant that there was no significant difference in mean incidence rates between the districts overall (H = 25.978, p = 0.354); however, the highest mean incidence rates were found in districts in the west of the state with relatively low human population density, including Kuala Penyu, Nabawan, Tenom and Kota Marudu. The relative risks were highest in Kuala Penyu, Kota Marudu and Kudat districts (RR = 3.5, 2.1 and 1.8, respectively) (Table 1). The 4 highest-incidence districts reported a low proportion of cases residing in urban localities (0–12%) (Table 1). Lower, less variable incidence rates were recorded from some of the central and eastern districts including Kinabatangan, Tongod, Kunak and Tawau (annual incidence range of 3–62 cases/100,000 each year). These districts also had some of the lowest relative risks (RR = 0.3, 0.4, 1, and 0.8, respectively) (Table 1), along with a wide range in proportions of urban cases (4–73%).

**Fig 5:**
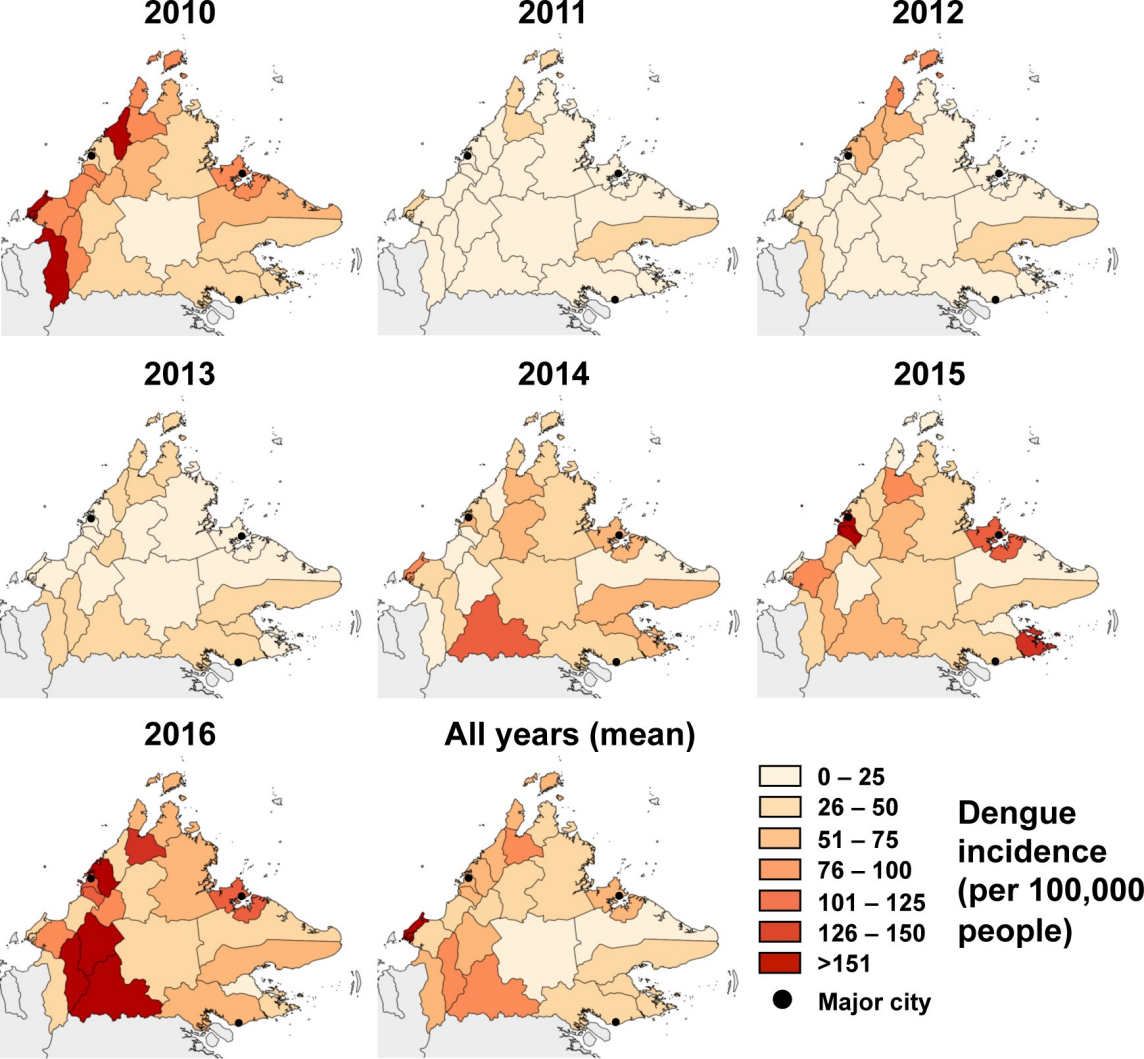
Annual spatial incidence of dengue in Sabah, 2010–2016. Incidence rates across districts are shown for each year, as well as the overall mean annual incidence during the 7-year period. The 3 major cities of Sabah (Kota Kinabalu, Sandakan and Tawau) are indicated by black points.

The changing annual spatial trend is shown in Fig 5, which indicates high annual variability across districts, and the highest mean incidence rates overall in the western districts of Sabah. A spatial shift occurred during the large 2015 outbreak, when incidence increased markedly in the more densely populated western districts of Kota Kinabalu, Penampan, and Putatan, as well as in Sandakan and Semporna in the east (Fig 5). The overall urban case proportions in these districts ranged from 53–85%. During 2016, cases from both urban and rural localities contributed to the outbreak, but the greatest overall burden was in rural localities of Tenom, Nabawan and Keningau districts.

### Severe dengue

**Fig 6:**
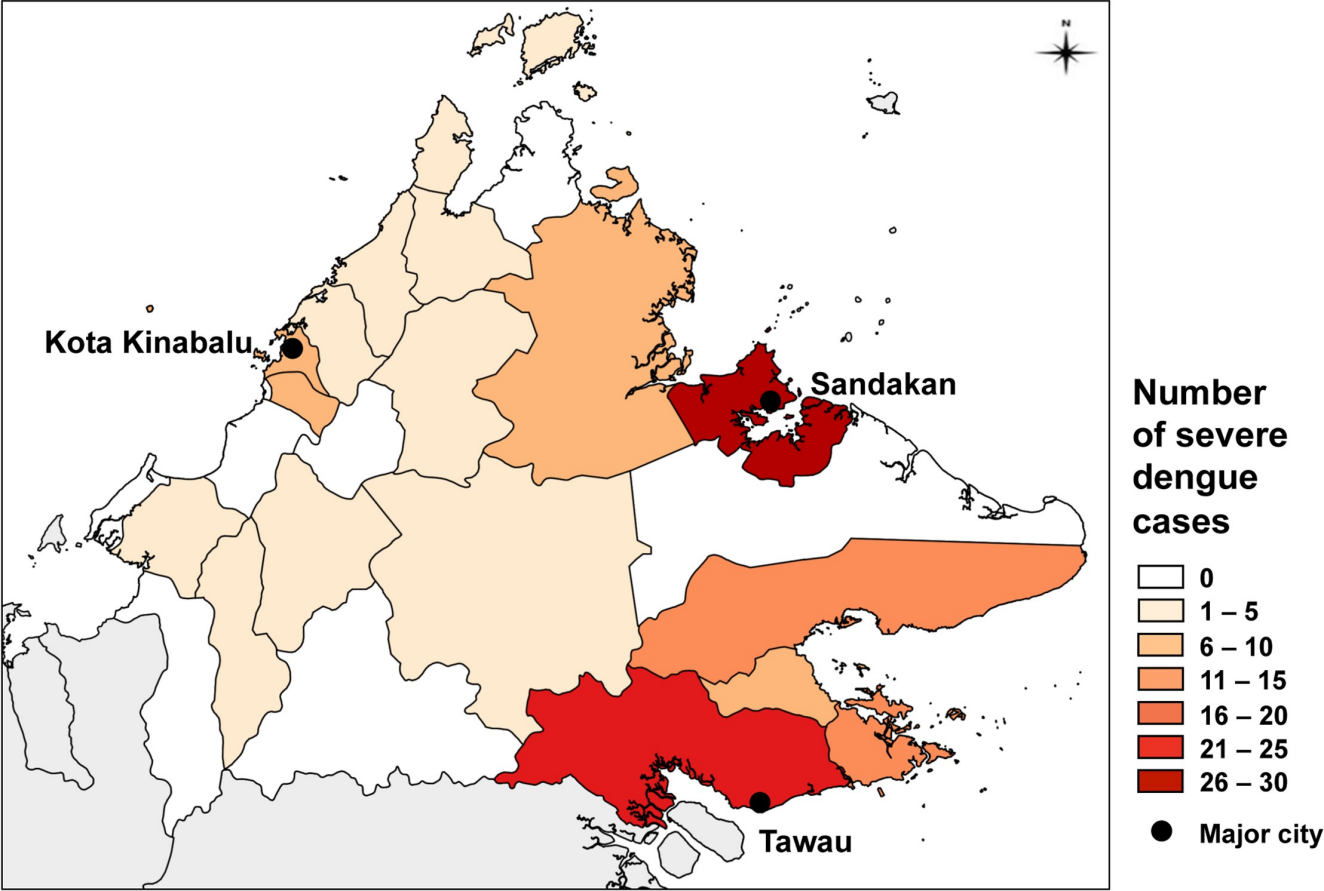
Total severe dengue notifications by district, 2010–2016. Total number of severe dengue cases reported for each district during the 7-year period. The 3 major cities of Sabah (Kota Kinabalu, Sandakan and Tawau) are indicated.

Of all dengue cases reported over the 7 years, 1.1% were severe (haemorrhagic) dengue cases. Severe cases were reported across all years, with an average of 18 severe cases/year across the state. The greatest proportion of severe cases were concentrated in Sandakan (24%) and Tawau (20%) districts on the eastern side of the state, and this was consistent across all years. The highest severe dengue incidence occurred in Kunak, Sandakan and Tongod, while the lowest was observed in the western districts, several of which recorded zero severe cases despite recording high overall dengue incidence (Figs 5 and 6, Table 1). Severe dengue occurred in expected proportions relative to population size of both genders and age groups, with the highest burden (35%) reported in the 10–19 years age group. There were 9 severe dengue deaths during the study period, 4 of which were in Tawau district.

### Entomological factors

Entomological data (House Indices) collected during the 2015–2016 outbreaks indicated that the partially sylvatic vector *Aedes albopictus* was the predominant species identified from larval collections in and around rural and urban case residences (Table 2). Of 719 dengue case residences that were inspected as part of active surveillance in 2015–2016, 618 were found to contain mosquito larvae (HI = 86%). Of those, *Ae*. *albopictus* larvae were identified from 394 residences (HI = 64%), either alone (383 residences) or with *Ae*. *aegypti* (11 residences). Conversely, 94 residences were positive for *Ae*. *aegypti* (83 alone, 11 with *Ae*. *albopictus;* HI = 15%), 33 residences were positive for *Culex* species (HI = 5%), and 108 samples could not be identified (17%).

The specific districts where case residences were inspected are detailed in S1 Table. Seven hundred and nineteen inspections were conducted in 21/25 districts, with the greatest number performed in Tawau (158 inspections; 96 of which were in urban localities) and Nabawan (107 inspections; all in rural localities). The residences with the highest proportions of *Ae*. *aegypti* were in the east coast districts of Tawau (larvae found in 60/158 residences) and Lahad Datu (18/43 larvae-positive residences). *Ae*. *albopictus* was the most prevalent species across both urban and rural residences of most of districts surveyed.

## Discussion

### Spatial and temporal trends

In recent decades, the scale of epidemics in South East Asia has increased, including in the Malaysian state of Sabah, where incidence rates have increased substantially since 2014. While spatial trends varied from year to year, the highest incidence across all years occurred in districts along the western coast of the state. The timing of large epidemic years in Sabah (2010, 2015 and 2016) was consistent with patterns observed at national and regional levels during the same period (wider Malaysia, Indonesia, Philippines) [11, 38]. This suggests shared climatic influences on outbreak occurrence. Across the tropics, dengue transmission occurs all year round with temporal peaks and troughs whose causative relationship with temperature and rainfall remains poorly defined [39, 40].

Our observations are derived from district-level incidence data and we were not able to evaluate finer scale incidence patterns. However, we were able to illustrate marked spatial and temporal heterogeneity across Sabah in all years and noted a distinct shift in the spatial dominance of urban versus rural localities during the large outbreak of 2015, which reversed again in 2016. Our findings suggest that high density, urbanised areas are not necessarily the primary locations of ongoing epidemics in Sabah. Similar observations of rural dominance or equivalence in dengue incidence have also been observed in other areas of Malaysia [16, 41] and elsewhere in the region, including in Cambodia, Thailand, Vietnam and Sri Lanka [42–46]. These latter countries have all reported epidemics that shift between rural and urban areas via human or mosquito movement. In endemic environments, transmission is mediated by water storage and waste disposal practices, mosquito vector ecology and sociocultural factors including human movement patterns [47–51]. The relative importance of these is still not well understood [52, 53]. In Sabah, effectively addressing these risks will require dengue management strategies to be applied across a range of ecological settings.

### Demographic factors

Our findings indicated that the age-related dengue risk in Sabah was in line with regional trends indicating a transition from children to adults being disproportionately affected by dengue [54]. Incidence was higher for males than for females across all districts of the state, and was significantly higher for both genders in the 10–29 age group. This higher risk may suggest that a large proportion of people in this age-group (and males in particular) were either engaged in outdoor activities or possibly being occupationally exposed to mosquitoes. Although exposure to dengue vectors can occur indoors, outdoor activities, especially those in close proximity to forests or forest edges, are thought to increase the risk of being bitten by the abundant exophilic vector, *Ae*. *albopictus* [55, 56].

The agriculture sector is the major employment sector in Sabah. Living or working in proximity to rubber plantations or forested areas has been linked to increased vector-borne disease risk, in Sabah and in other parts of South East Asia [57, 58]. This is due to changing human interactions with particular land use types, where vector and host ecology have been altered [9, 55, 59]. The particularly high proportion of males affected in the largely rural Tongod and Kinabatangan districts may reflect a greater proportion of men engaged in occupational or recreational outdoor activities. Further investigation of employees of outdoor occupations, such as agricultural workers, or of residential populations close to agricultural or forested areas of Sabah, could shed light on the specific demographic factors and land use characteristics associated with infection.

### Severe dengue

Changing demographic or immunological factors may explain the observed pattern of severe dengue in our study. Incidence of severe dengue was localised to two main regions of the state: the eastern districts of Tawau and Sandakan. These districts include major urbanised cities as well as rural surrounding areas, and comprised relatively low dengue rates compared to the west of the state. The reasons for this spatial concentration of cases in these eastern districts is unknown. It is possible that the increase in severe cases in these areas followed a serotype switch from DENV 4 to DENV 1 reported to have occurred in Sandakan between 2013 and 2016 [26]. Major dengue outbreaks commonly follow the switching of DENV serotypes and the subsequent loss of herd immunity in the human population. Those with a history of dengue infection with a different serotype may be more vulnerable to severe dengue as a result of antibody-dependent enhancement of infection [60–62]. Surveillance information regarding which virus serotypes and genotypes were circulating in Sabah was not available in this study, so we were unable to assess the potential contribution of virus circulation patterns to the trends we observed. However, routine public health monitoring of circulating serotypes, including the importation of new serotypes in particular, should be prioritised. Close monitoring of serological surveillance data alongside detailed epidemiological data could aid predictions of severe disease risk [63, 64].

### Entomological factors

*Ae*. *albopictus* was by far the commonest potential dengue vector identified by the public health authorities. It was three times more common than *Ae*. *aegypti* in urban case households and seven times more common in rural households. In >50% of case households inspected, *Ae*. *albopictus* was the only potential vector identified. The eastern districts of Sabah state appeared to have a higher proportion of *Ae*. *aegypti* compared to the rest of the state, although overall dengue incidence was lower on the east coast. This finding was consistent with those of early entomological surveys of Sabah in the 1970’s, which reported higher numbers of *Ae*. *aegypti* on the east coast and lower abundance on the west coast [65, 66]. In those studies, the greater presence of *Ae*. *aegypti* in the east was thought to be due to more frequent travel by boat between east coast settlements for fishing and trade. Although *Ae*. *aegypti* is generally considered responsible for most dengue transmission in South East Asia [67, 68], *Ae*. *albopictus* is more common than *Ae*. *aegypti* across Malaysia and Borneo [55, 56, 59, 69]. Our results are consistent with these previous surveys, and indicate that this species was dominant during the two largest and most recent dengue outbreaks in our study period.

The presence of natural and artificial larval habitats for *Ae*. *albopictus* have previously been associated with epidemics in both urban and rural areas of Malaysia [70–72]. Urban dominance of *Ae*. *albopictus* has also been observed, at least seasonally, in parts of Thailand, southern China and other South East Asian countries [73–75]. Vector incrimination studies for Malaysia are rare, but Ming *et al*. showed that *Ae*. *albopictus* was 3–10 fold more abundant than *Ae*. *aegypti* around Kuala Lumpur and 3–5 times less likely to harbour dengue virus [76]. The potential role of *Ae*. *albopictus* in transmission in Sabah, and how that might be affected by human density and biting preference in rural areas, requires further investigation.

### Limitations

The revised dengue case definition in Malaysia in 2014 will undoubtedly have influenced the incidence rates reported here, in terms of either under- or over-reporting of cases. Less reliance on clinical symptoms for case notification from 2014 onwards might have been expected to reduce notifications but, in fact, a dramatic increase in cases was recorded. Perhaps the influx of diagnostic kits into health facilities across the country encouraged testing of all febrile cases, accompanied by changes in awareness and reporting by clinicians. Prior to 2014, there may have been a lack of resources for testing or notifying dengue, or other socioeconomic factors, that resulted in under-reporting [77]. It is also possible that increases in diagnostic testing from 2014 were not uniform across all districts, and/or that additional reporting inconsistencies at the sub-district level, or between rural and urban health facilities, may have impacted our observations. However, widespread increases in cases recorded throughout the rest of the country during the same time frame suggest that much of the true dengue burden was not being captured prior to 2014. Longer-term monitoring of notification trends will likely clarify the true burden. Similarly, finer-scale investigation at the sub-district level might enable more detailed analyses of dengue risk factors, as variation in population density and demographic factors within districts could be more accurately assessed. Additional immunological, biological and/or virological factors may also have impacted our findings, but an assessment of these was beyond the scope of this study.

The use of house index (HI, percentage of houses positive for mosquito larvae) for entomological surveillance is not a good correlate of dengue infection risk, but it remains one of the most widely used entomological indices of operational programs. Larval surveys are, however, appropriate to survey for the presence/absence of potential vectors. In this study, the HIs for *Ae*. *aegypti* and *Ae*. *albopictus* in Sabah indicate that *Ae*. *albopictus* is extremely common around dengue case houses, often in the apparent absence of *Ae*. *aegypti*. It remains possible that *Ae*. *aegypti* was simply more cryptic than *Ae*. *albopictus* and that, despite its apparent absence, it was the key vector. Nonetheless, the relative roles of *Ae*. *albopictus* and *Ae*. *aegypti* as vectors in Sabah are important to define through further vector incrimination studies.

### Future directions

Our study describes spatial, temporal, demographic and vector-related characteristics of dengue disease patterns in Sabah state. To further explain some of the trends observed, finer-scale collection of demographic data, and additional field investigations are necessary. In particular, surveys of socioeconomic variables associated with dengue, including human movement in urban and rural landscapes, would aid identification of high-risk groups. Further investigations and monitoring of the spatial and temporal movement of virus serotypes and vectors could also inform prevention strategies. Ultimately, linking both vector and serological surveillance to the dengue case notification system would enhance existing public health efforts. Considering the ongoing expansion of dengue endemicity and burden in the region, proactive strategies to increase understanding of the complex and evolving epidemiological factors underlying dengue risk across varied environments are critical.

## Supporting information

S1 Checklist STROBE checklist for observational studies. (DOCX)

S1 Fig Seasonal decomposition of incidence rates in Sabah, 2010–2016. The seasonal trend of dengue is shown in panel A, with the largest seasonal peak occurring on average between Nov and May each year (indicated by vertical black lines). The additional components separated from the seasonal trend during the decomposition procedure are also indicated in panels B-D (cyclical component (B), irregular component (C) and overall smoothed trend (D)).
(TIF)

S2 Fig Variation in dengue incidence across Sabah districts, 2010–2016. Dengue mean monthly incidence rates are plotted a) for the years 2010–2013, and b) during 2014–2016. The monthly mean (line), range (upper and lower whiskers), and outlying values are indicated for each district.
(TIF)

S1 Table Entomological surveillance of case residences by district, 2015–2016. (DOCX)
