# Supplementary figures and images for: Incidence and epidemiological features of dengue in Sabah, Malaysia
Source: PLoS Negl Trop Dis. 2020 May 11;14(5):e0007504. doi: 10.1371/journal.pntd.0007504 (PMC7241834; doi:10.1371/journal.pntd.0007504)

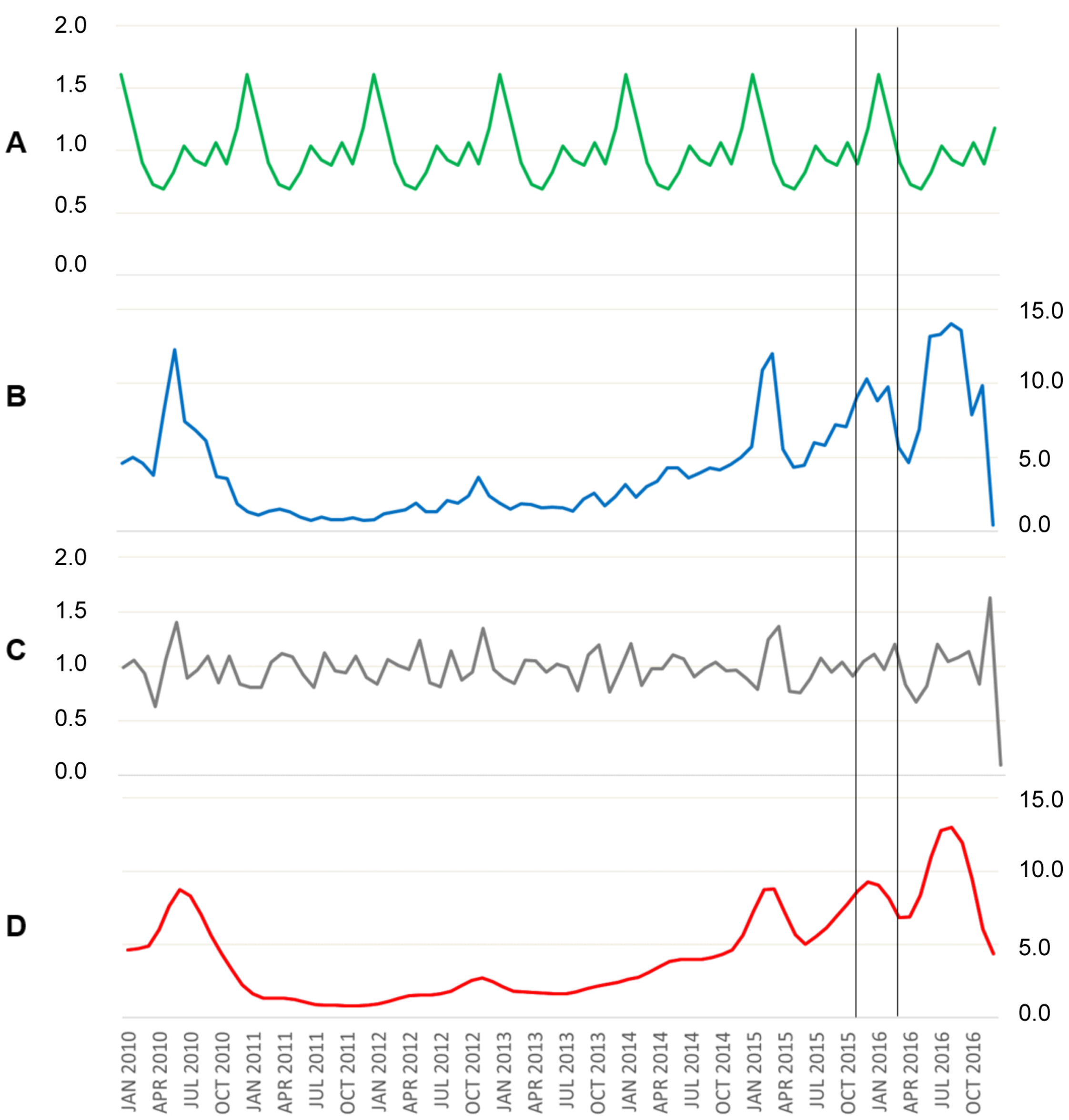

Supplement: S1 Fig — The seasonal trend of dengue is shown in panel A, with the largest seasonal peak occurring on average between Nov and May each year (indicated by vertical black lines). The additional components separated from the seasonal trend during the decomposition procedure are also indicated in panels B-D (cyclical component (B), irregular component (C) and overall smoothed trend (D)). (TIF) [file pntd.0007504.s002.tif]

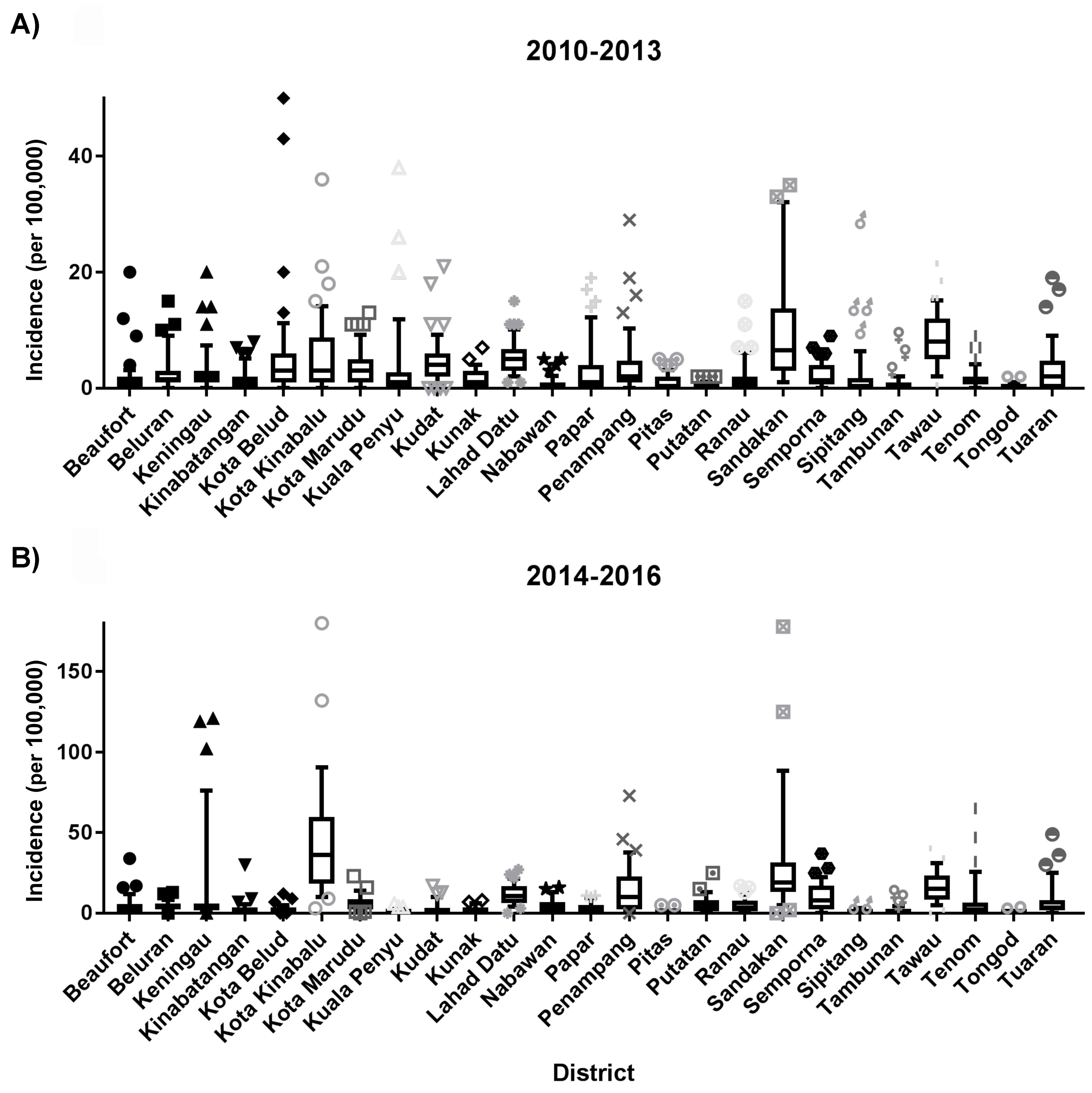

Supplement: S2 Fig — Dengue mean monthly incidence rates are plotted a) for the years 2010–2013, and b) during 2014–2016. The monthly mean (line), range (upper and lower whiskers), and outlying values are indicated for each district. (TIF) [file pntd.0007504.s003.tif]
