# Supplementary material for: Incidence and epidemiological features of dengue in Sabah, Malaysia
Source: PLoS Negl Trop Dis. 2020 May 11;14(5):e0007504. doi: 10.1371/journal.pntd.0007504 (PMC7241834; doi:10.1371/journal.pntd.0007504)
Supplement: S1 Table — (DOCX) [file pntd.0007504.s004.docx]

### S3 Table. Case residence inspections by district, 2015-2016.

| **Dengue case residence inspections** | | | | | | **Number of larvae-positive residences with specific species present** | | | | |
| --- | --- | --- | --- | --- | --- | --- | --- | --- | --- | --- |
| **District** | **Urban residences** | **Rural residences** | **Unspecified residences** | **Total inspected** | **Total larvae-positive residences (HI)** | ***Ae. aegypti*** | ***Ae. albopictus*** | ***Ae. aegypti* & *Ae. albopictus*** | ***Culex* spp.** | **Undetermined** |
| Beaufort | 0 | 1 | 0 | 1 | **1 (1.0)** | 0 | 1 | 0 | 0 | 0 |
| Beluran | 3 | 5 | 1 | 9 | **9 (1.0)** | 1 | 3 | 0 | 0 | 5 |
| Keningau | 12 | 27 | 8 | 47 | **47 (1.0)** | 1 | 46 | 0 | 0 | 0 |
| Kinabatangan | 0 | 1 | 0 | 1 | **1 (1.0)** | 0 | 1 | 0 | 0 | 0 |
| Kota Belud | 0 | 56 | 3 | 59 | **58 (0.98)** | 0 | 0 | 0 | 0 | 58 |
| Kota Kinabalu | 30 | 11 | 0 | 41 | **17 (0.42)** | 2 | 13 | 0 | 2 | 0 |
| Kota Marudu | 0 | 22 | 1 | 23 | **23 (1.0)** | 0 | 22 | 0 | 1 | 0 |
| Kuala Penyu | 0 | 0 | 0 | 0 | **0** | 0 | 0 | 0 | 0 | 0 |
| Kudat | 19 | 21 | 2 | 42 | **29 (0.69)** | 4 | 21 | 1 | 0 | 3 |
| Kunak | 4 | 0 | 0 | 4 | **1 (0.25)** | 0 | 0 | 0 | 0 | 1 |
| Lahad Datu | 19 | 27 | 0 | 46 | **43 (0.94)** | 12 | 21 | 6 | 2 | 2 |
| Nabawan | 0 | 107 | 0 | 107 | **107 (1.0)** | 0 | 46 | 0 | 22 | 39 |
| Papar | 7 | 21 | 0 | 28 | **28 (1.0)** | 0 | 28 | 0 | 0 | 0 |
| Penampang | 41 | 34 | 2 | 77 | **58 (0.75)** | 1 | 51 | 1 | 5 | 0 |
| Pitas | 1 | 0 | 0 | 1 | **1 (1.0)** | 0 | 1 | 0 | 0 | 0 |
| Putatan | 8 | 23 | 0 | 31 | **11 (0.36)** | 0 | 11 | 0 | 0 | 0 |
| Ranau | 0 | 0 | 0 | 0 | **0** | 0 | 0 | 0 | 0 | 0 |
| Sandakan | 1 | 0 | 0 | 1 | **1 (1.0)** | 0 | 1 | 0 | 0 | 0 |
| Semporna | 0 | 0 | 0 | 0 | **0** | 0 | 0 | 0 | 0 | 0 |
| Sipitang | 0 | 0 | 1 | 1 | **1 (1.0)** | 0 | 1 | 0 | 0 | 0 |
| Tambunan | 0 | 0 | 0 | 0 | **0** | 0 | 0 | 0 | 0 | 0 |
| Tawau | 96 | 58 | 4 | 158 | **158 (1.0)** | 57 | 98 | 3 | 0 | 0 |
| Tenom | 2 | 1 | 3 | 6 | **6 (1.0)** | 0 | 6 | 0 | 0 | 0 |
| Tongod | 0 | 1 | 0 | 1 | **1 (1.0)** | 0 | 0 | 0 | 1 | 0 |
| Tuaran | 12 | 21 | 2 | 35 | **17 (0.49)** | 5 | 12 | 0 | 0 | 0 |
| **Total** | **255** | **437** | **27** | **719** | **618 (0.86)** | **83 (0.13)** | **383 (0.62)** | **11 (0.02)** | **33 (0.05)** | **108 (0.17)** |

HI = proportion of larvae-positive houses out of all inspected houses. ‘Undetermined’ = larvae whose identification was incomplete or pending.
